# Assembly of Natively Synthesized Dual Chromophores Into Functional Actinorhodopsin

**DOI:** 10.3389/fmicb.2021.652328

**Published:** 2021-04-28

**Authors:** Kimleng Chuon, So Young Kim, Seanghun Meas, Jin-gon Shim, Shin-Gyu Cho, Kun-Wook Kang, Ji-Hyun Kim, Hyun-Suk Cho, Kwang-Hwan Jung

**Affiliations:** ^1^Department of Life Science and Institute of Biological Interfaces, Sogang University, Seoul, South Korea; ^2^Research Institute of Basic Sciences, Seoul National University, Seoul, South Korea

**Keywords:** actinobacteria, microbial rhodopsin, light-driven proton pumps, retinal, carotenoid, dual chromophore actinorhodopsin

## Abstract

Microbial rhodopsin is a simple solar energy-capturing molecule compared to the complex photosynthesis apparatus. Light-driven proton pumping across the cell membrane is a crucial mechanism underlying microbial energy production. *Actinobacteria* is one of the highly abundant bacterial phyla in freshwater habitats, and members of this lineage are considered to boost heterotrophic growth *via* phototrophy, as indicated by the presence of actino-opsin (ActR) genes in their genome. However, it is difficult to validate their function under laboratory settings because *Actinobacteria* are not consistently cultivable. Based on the published genome sequence of *Candidatus aquiluna* sp. strain IMCC13023, actinorhodopsin from the strain (ActR-13023) was isolated and characterized in this study. Notably, ActR-13023 assembled with natively synthesized carotenoid/retinal (used as a dual chromophore) and functioned as a light-driven outward proton pump. The ActR-13023 gene and putative genes involved in the chromophore (retinal/carotenoid) biosynthetic pathway were detected in the genome, indicating the functional expression ActR-13023 under natural conditions for the utilization of solar energy for proton translocation. Heterologous expressed ActR-13023 exhibited maximum absorption at 565 nm with practical proton pumping ability. Purified ActR-13023 could be reconstituted with actinobacterial carotenoids for additional light-harvesting. The existence of actinorhodopsin and its chromophore synthesis machinery in *Actinobacteria* indicates the inherent photo-energy conversion function of this microorganism. The assembly of ActR-13023 to its synthesized chromophores validated the microbial community’s importance in the energy cycle.

## Introduction

Actinobacteria are found in various terrestrial and aquatic environments. They are the most morphologically diverse prokaryotes (Servin et al., 2008). The unique genes and novel metabolic pathways present in the organism are beneficial for natural adaptation. Also, marine *Actinobacteria* are distributed in various marine habitats, such as marine sediments (Claverías et al., 2015), seawater (Subramani and Aalbersberg, 2013), freshwater ecosystems (Allgaier and Grossart, 2006), and melting sea ice (Fernández-Gómez et al., 2019). The *Actinobacteria* clade (OM1 clade; Rappé et al., 1998), constituted by members of the class Actinobacteria, had been recognized as a contributor to marine microbial communities in culture-independent studies, which involved 16S rRNA gene pyrosequencing, metagenome sequencing, and metaproteomic experiments (Jensen and Lauro, 2008). The *Actinobacteria* strain IMCC13023 was isolated from a surface seawater sample collected from Kongsfjorden (Svalvard, Norway) during the glacier-melting season using high-throughput extinction-to-dilution culturing. The strain was classified under the current name *Candidatus Aquiluna* sp. IMCC13023, candidatus name (Cho and Giovannoni, 2004; Kang et al., 2012). A protein-centric comparative metaproteomic approach adopted on an oceanic scale, targeting membrane proteins, suggested the involvement of the bacteria in nutrient transport and energy transduction, which altered our understanding of the bacterial community structure, nutrient utilization, and energy transduction, as well as viral and archaeal activities (Morris et al., 2010).

Light is a source of energy and an environmental cue that available in excess in most surface environments. In prokaryotic systems, it has been utilized by photoautotrophs and photoheterotrophs. However, the conversion of light to cellular energy has been characterized in only a few species. Freshwater actinobacteria are ubiquitous in light-illuminated aquatic environments and grow more rapidly in the absence of functional photosystems, likely because sugar transport is upregulated in light (Maresca et al., 2019). In a previous report, the actinobacterium *Rhodoluna planktonica* was shown to encode one actinorhodopsin (RpActR), and the functional expression of RpActR was demonstrated in native cells. Illumination induced the acidification of actinobacteria cell culture and elevated the cellular ATP content, indicating ActR-based phototrophy in native actinobacteria (Nakamura et al., 2016). Rhodopsins found in Archaea, Bacteria, and Eukaryotes, which contained opsin and a covalently bound retinal as a chromophore to absorb photons for energy conversion or the initiation of intra or intercellular signaling (Spudich et al., 2000). Microbial communities utilized the microbial rhodopsin or typed I rhodopsin with all-trans-retinal for various purposes. At the same time, animals solely used a different rhodopsin family with 11-cis retinal for visual and nonvisual phototransduction, classified as type II rhodopsin (Palczewski, 2006; Shichida and Matsuyama, 2009). Rhodopsins had been divided for microbial and animal rhodopsin, also known as type-I and type-II rhodopsin, respectively (Ernst et al., 2014). The gene for actinorhodopsin, a type I rhodopsin-family protein previously detected in actinobacteria isolates, was also found in the strain IMCC13023 (Sharma et al., 2008, 2009). Here, we demonstrated that ActR-13023 exhibited phototrophy in native actinobacterium, which confirmed the functional expression of the ActR-13023 gene, and light illumination induced the acidification of the actinobacterial cell culture. ActR-13023 expressed heterologously in *Escherichia coli* facilitated accurate characterization, and the findings suggested the assembly of this actinorhodopsin with retinal/carotenoid as a light-harvesting protein.

## Materials and Methods

### Molecular Cloning

*Candidatus Aquiluna* sp. IMCC13023 cells were generously provided by Jang-Cheon Cho from the Department of Biology, Inha University, Korea. PCR primers with restriction enzyme sites and his-tag sequences were used for colony PCR. The expression vector pKA001 (Pushkarev et al., 2018) under the IPTG inducible promoter was used to express the ActR-13023 gene. pACK-*blh* was used to express the β-carotene dioxygenase gene, and pACK-ctrw7120 was used to expressing the β-carotene ketolase genes from *Nostoc* sp. PCC7120 (for canthaxanthin production; Jang et al., 2011; Yang and Guo, 2014; Gao et al., 2020). The expression vector for carotenoid was successfully constructed with an addition of a multiple cloning site under an inducible promoter into an original pAC-BETA plasmid (Addgene, United States). The detailed information of primers sequences and a global map of the expression vectors with restriction enzyme sites were documented in supplementary data (Supplementary Figure S1).

### Protein Expression and Purification

*Escherichia coli* strain UT5600 was used for the expression system. The cloned plasmid, containing the recombinant ActR-13023 sequence, was transformed into *E. coli*. To express these proteins, the transformed *E. coli* cells were cultured in LB media until OD_600nm_ = 0.4. Then, protein expression was achieved by inducing 1 mM of IPTG (Applichem, United States) and 5 μM all-*trans*-retinal (Sigma, United States), and culture for 5 h at 37°C with shaking at 240 rpm. The cells were collected and sonicated (Branson Sonifier 250, United States), and the membrane fraction was collected by ultracentrifugation for 1 h at 35,000 × *g*. The membrane fraction was mixed with 2% n-dodecyl-*β*-D-maltopyranoside (DDM) in the same volume to obtain a final solution of 1% DDM (Anatrace, United States), and the sample was continuously inverted in a chamber at 4°C for 4 h. After detergent solubilization, the sample was centrifuged at 20,000 × *g* for 15 min at 4°C to separate solubilized protein from the cell membrane. Then, the supernatant was collected and treated with 1 ml volume of Ni^+2^-NTA resin (Qiagen, Korea). Immobilized metal affinity chromatography (IMAC) for the His-tag fusion protein technique was applied to extract the pure target protein. The protein bound to the Ni^+2^-NTA resin was loaded to the affinity column, and the sample was washed with 25 mM imidazole (Sigma) in 0.02% DDM twice to remove non-specific binding random histidine. Finally, the target protein was eluted with 250 mM imidazole in 0.02% DDM, and the sample was filtered by Amicon centrifugal filter (Merckmilliphore, Germany). The eluted protein was washed and concentrated through Amicon centrifugal filter with a 10 kDa molecular weight cutoff membrane. The sample was washed using 5 ml of 0.02% DDM buffer to push through, and concentrated sample to 0.3⁓0.5 ml and then resuspended with another 5 ml of the same buffer to continuously repeat the process three times. Purified rhodopsin showed purple color in the 0.02% DDM solution, and the SDS-PAGE of the purified sample was assessed to confirm the protein’s molecular mass and purity. Purified rhodopsin was kept in 0.02% DDM at 4°C.

### Absorption Spectroscopy and pKa Measurements

UV/VIS spectrophotometer (Shimadzu, UV-2550) was used to measure the absorbance spectra of purified ActR-13023 in DDM solution. To measure the entire spectrum, 0.02% DDM solution was used for baseline, and the sample was measured every 0.5 nm wavelength from 350 to 750 nm. The absorption spectra of wild-type and mutants in DDM were recorded in various pH conditions. Samples were divided into two sets, and one set started with pH 7.0 down to pH 4.0 with a 0.5 pH change that was adjusted by adding a tiny amount of HCL. The other set with the same condition started from pH 7.0 up to pH 10.0 with a 0.5 pH change adjusted by adding a tiny NaOH amount. Together, we collected the spectroscopic titration data of ActR-13023 wild-type and mutants dependent on pH changes. The maximum absorption of microbial rhodopsin is shifted upon protonated and deprotonated states of the Schiff base (SB) counter-ion as previously described (Spassov et al., 2001). The data were fitted with a function containing pKa component as the following equation $y=A/\left(1+10^{pH-pKa}\right)$ where *A* is maximum absorbance changes in Origin Pro 7.0.

Hydroxylamine reactions were carried out at pH 7.0 in a mixture of 10 mM Tris-HCL (using a small amount of HLC to adjust Tris containing pH to 7.0), 0.1% DDM, 150 mM NaCl, and 0.2 M hydroxylamine. The sample was stirred continuously along with white light irradiation at an intensity of 100 W/m^2^ using a short wavelength cutoff filter (>440 nm, Sigma Koki SCF-50S-44Y, Japan) for 30 min. After the reaction, the cell sample was collected by centrifuge at 4,000 rpm at 4°C for 15 min, following cell wash with 15% ethanol one time then collected in the same condition.

### Proton Pumping Measurements

Proton pumping experiments were performed using whole-cell and spheroplast vesicles, as previously described (Zhu et al., 2013; Maresca et al., 2016). The spheroplast vesicles were isolated by centrifugation at 30,000 × *g* for 1 h at 4°C (Beckman XL-90 ultracentrifuge) and washed by 3 ml of 10 mM NaCl, 10 mM MgSO_4_.7H_2_O, and 100 μM CaCl_2_ (Wang et al., 2003). About 30 μl of carotenoid in ethanol with maximum absorbance density at 3 units (OD = 3) was added to 3 ml of ActR-13023-expressing spheroplasts by vigorously stirring in 50 mM MgSO_4_ and 50 mM Na-EDTA for an additional 30 min, followed by further incubation for 16 h at 4°C, washing twice with sonication buffer (50 mM Tris, 150 mM NaCl, pH 7), and resuspension in an unbuffered solution (10 mM NaCl, 10 mM MgSO_4_.7H_2_O, and 100 μM CaCl_2_). The samples were maintained in an unbuffered solution, and the pH of the solution was recorded. The sample solution was illuminated at an intensity of 100 W/m^2^ using a short wavelength cutoff filter (>440 nm, Sigma Koki SCF-50S-44Y, Japan) in combination with a focusing convex lens and heat protecting (CuSO_4_) filter, and the pH values were monitored using a Horiba pH meter F-51. The sample was stored in the dark for 5 min, and the pH values were measured in the initial dark state followed by illumination for 3 min and 3 min of darkness after illumination, and 10 μM of carbonyl cyanide m-chlorophenyl hydrazine (CCCP) was used in CCCP treated experiments. Proton change was converted by 10^−ΔpH^ and then multiply with 10^pH^ (initial pH), and the average data were calculated and fitted using Origin Pro 7.0.

### Light-Induced Differential Spectroscopy and Photocycle Measurements

The purified ActR-13023 wild-type and mutants were stored in 0.02% DDM, and then had been measured by the time-based kinetic function of Scinco spectrophotometer (Scinco, Korea) upon light illumination. The samples had been prepared in 1 ml volume cuvette at 0.5 OD and kept in the dark conditions for 10 min at 4°C. The dark-adapted samples were used for baseline spectrum; then, the samples were illuminated with white light at an intensity of 100 W/m^2^ guided by an optical tube for 1 min. The light-induced absorption differences were measured every 700 msec for 60 times, and the signals were averaged as one data. Three experiments were repeated and fitted the overall differential spectra in Origin Pro 7.0.

Sample preparation and experimental set-up for photocycle had been performed by flash-induced transient absorption changes using RSM 1000 spectrophotometer (Olis, United States). ActR-13023 expressed cells were sonicated (Branson Sonifier 250, United States) three times at 45% amplitude for 2:30 min in 30-s intervals. The sample was centrifuged to check for cell lysis at 4,000 rpm at °C for 15 min, and the supernatant was collected. The pellet containing the unbroken cell was discarded. The broken membrane fragments were then collected with ultracentrifuge at 20,000 rpm at 4°C for 1 h. Flash-photolysis experiments were performed on the membrane fragments encased within polyacrylamide gel. Membrane fragments were resuspended in 0.4 ml of pH 8.0 (50 mM Tris and 150 mM NaCl), and then added 0.3 ml of 33% acrylamide and 1% bis-acrylamide solution (Dynebio, Korea), 2.4 μl of 10% ammonium persulfate (AP), and 3 μl of TEMED (N, N, N′, N′ tetramethylethylenediamine), which was used to study photocycle of microbial rhodopsin to the closest native form in *E. coli* membrane previously (Miranda et al., 2009). The samples were placed in plastic cases that mimic the cuvette shape and size. After solidification, the gel was removed and washed with deionized water (DIW) for 4 h at 23°C and store at 4°C for the next experiment. The samples were soaked in pH 7–8 buffer solution (50 mM Tris and 150 mM NaCl), pH adjustment using HCL solution. The experiment was conducted by a custom-built single-wavelength spectrometer described before (Waschuk et al., 2005). In short, the photocycle experiment started with 6 ns pulses of an Nd-YAG pulse laser (Continuum, Mini-light II, 532 nm, 6 ns, 25 mJ), and optical filters were applied for selected wavelengths. At least 30 signals were averaged for each data points before the fitting process.

### Carotenoid Interaction Measurement

*Salinibacter ruber* was used to isolate salinixanthin, and the cells were grown at 37°C in a shaking incubator (180 rpm) in 200 ml Erlenmeyer flasks containing 50 ml medium of the following composition NaCl, 195; MgSO_4_.7H_2_O,25; MgCl_2_6H_2_O,16.3; CaCl_2_2H_2_O,1.25; KCl,5.0; NaHCO_3_,0.25; NaBr,0.625; yeast extract, 1.0 (all concentrations in g/L), and adjusted to pH 7.0 by HCL. Jang-Cheon Cho generously provided the actinobacteria strain IMCC13023, Inha University, Korea. The samples were collected and washed with DIW (10 ml). The cells were then sonicated (Branson Sonifier 250, United States) in DIW, and the cell lysis then mixes with methanol/acetone (3:7). The samples were centrifuged at 35,000 rpm, and the supernatant was taken to dryness. Then was dissolved in methanol and spotted on a silica gel 60 TLC plate (Sigma). The plate was developed by immersion in the corresponding solvents [100:1 ethyl acetate:ethanol and hexane (5:1 v/v)]. The carotenoid target samples were isolated from the plate and dissolved in petroleum ether, applied to the Symmetry C18 column (3.9 mm × 150 mm; Agilent, United States), and eluted with a mixture of acetonitrile and dichloromethane (31, v/v) at a flow rate of 1 ml/min. The product was confirmed based on high-resolution mass spectra data obtained from the Organic Chemistry Research Center, Sogang University, Orbitrap Mass Spectrometer, LTQ Orbitrap XL, Thermo Fisher Scientific, Korea.

The 3D structure prediction of ActR-13023 was modeled by automated protein structure homology SWISS-MODEL (PDB: 3ddl as a template), and an optimized PDB file was obtained for molecular docking prediction. Protein PDB file and carotenoid (canthaxanthin, PubChem ID: 5281227) were uploaded to an integrated docking server^1^ using the PM6 method by Mozyme function of MOPAC2009 as described by Bikadi and Hazai (2009). After each docking calculation, the RMSD between the lowest energy docked ligand, and the complex crystal structure was evaluated. Docking calculations were carried out using DockingServer. The MMFF94 force field (Kaminski and Jorgensen, 1996) was used for energy minimization of ligand molecule (***canthaxanthin***) using DockingServer. Gasteiger partial charges were added to the ligand atoms. Non-polar hydrogen atoms were merged, and rotatable bonds were defined. Docking calculations were carried out on the ***ActR-13023-optimized*** protein model. Essential hydrogen atoms, Kollman united atom type charges, and solvation parameters were added with AutoDock tools’ aid (Morris et al., 2009). Affinity (grid) maps of 20 × 20 × 20 Å grid points and 0.375 Å spacing were generated using the Autogrid program (Morris et al., 1998). AutoDock parameter set- and distance-dependent dielectric functions were used to calculate the van der Waals and the electrostatic terms, respectively. Docking simulations were performed using the Lamarckian genetic algorithm (LGA) and the Solis and Wets local search method (Solis and Wets, 1981). The initial position, orientation, and torsions of the ligand molecules were set randomly. Each docking experiment was derived from two different runs set to terminate after a maximum of 250,000 energy evaluations. The population size was set to 150. A translational step of 0.2 Å and quaternion and torsion steps of 5 was applied during the search.

## Results

### Features of ActR-13023 vs. Other Microbial Rhodopsins

**Figure 1:**
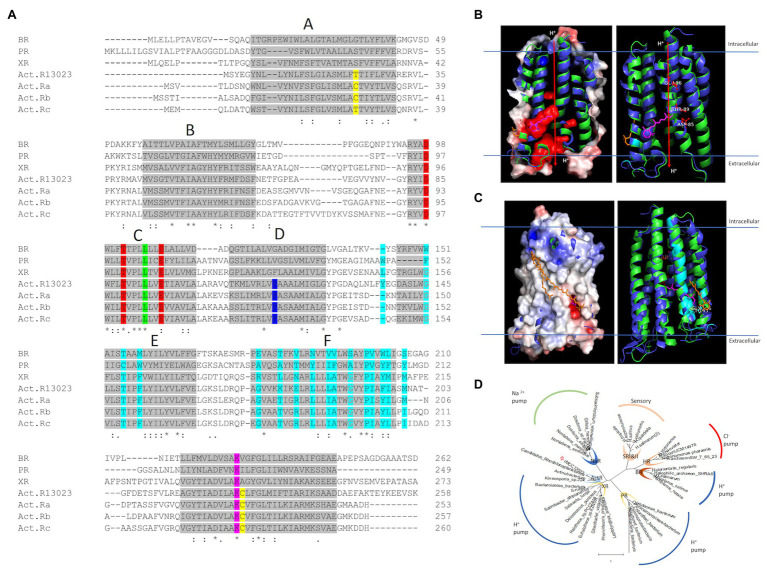
Features of ActR-13023 vs. other microbial rhodopsins. **(A)** Multiple sequence alignment of ActR-13023 with bacteriorhodopsin (BR), proteorhodopsin (PR), xanthorhodopsin (XR), and actinorhodopsin from acI actinobacteria clades A, B, and C (ActR a–c). Gray highlights and the alphabet represent the helical segment. Cys/Thr on helix A and G (disulfide bond in ActR) is marked in yellow, DTE motifs in red, color tuning residue in green, phylogenetically differentiated ActR XR, Pro in blue, carotenoid interaction residues in cyan, and retinal-bound Lys in pink. **(B)** Functional visualization of ActR-13023 compared to light-driven outward proton pump. **(C)** Carotenoid interaction potential of ActR-13023. **(D)** Phylogenetic tree of ActR-13023 vs. other microbial rhodopsins (the tree was generated using MEGA X 10.1).

Rhodopsin is a retinal-binding protein ubiquitous in unicellular microorganisms (Heberle et al., 2014). ActR-13023 from strain IMCC13023 contained all critical residues for function and secondary chromophore assembly. The newly discovered microbial rhodopsin’s photochemical and physiological characterization was essential to determine the protein’s potential as an important molecule in the microbial community. Proton pumps are associated with the most significant functional class of microbial rhodopsins and are widely distributed among microorganisms inhabiting a broad range of environments (Nakamura et al., 2016). The amino acid sequence comparison of ActR-13023 to bacteriorhodopsin (BR; Khorana et al., 1979), green-light absorption proteorhodopsin (PR; Bamann et al., 2014), xanthorhodopsin (XR; Luecke et al., 2008), and actinorhodopsin (ActR; Dwulit-Smith et al., 2018) revealed the DTE motif that associated to the proton-pumping process. ActR-13023 is a typical seven α-helical membrane protein with a chromophore, all-*trans*-retinal, covalently bound to lysine K224 (K216 in BR). The essential residues for proton transfer, aspartic acid D85 (D85 in BR; primary proton acceptor), and glutamic acid E96 (D96 in BR), were also conserved. Figure 1A showed the alignment of ActR-13023 to other microbial rhodopsin, including BR; however, the whole sequence of BR was used for the sequence alignment that why the position of D85 and D96 were not matched. Since the original position is different from the commonly used position reported by the crystal structure of BR using truncated form protein [complete sequence bacteriorhodopsin; Pinhassi et al., 2016; accession: WP_010903069]. The protein sequence alignment of ActR-13023 to BR, PR, and XR from *S. ruber* (PDB: 3ddl) that was reported for carotenoid interaction (Luecke et al., 2008), and other actinorhodopsins (Dwulit-Smith et al., 2018) showed that ActR-13023 shared 11 out of 18 residues that were reported for carotenoid interaction in XR. ActR-13023 had more conserved residues to XR than PR and BR, and the phylogenetic tree suggested a smaller distance to XR than the other microbial rhodopsin. A Pro residue in the middle of helix D is known to differentiate ActR from XR (Dwulit-Smith et al., 2018; Figure 1A). Structural alignment of modeling ActR-13023 to XR (PDB: 3ddl) helped to visualize the protein similarity, where electrostatic map revealed the negatively charged regions represented the DTE motifs of ActR-13023; each residue was marked in red and labeled (Figures 1A,B). The surface view of ActR-13023 with the carotenoid antenna-salinixanthin of XR revealed an interaction pattern, where Gly replaced a bulky residue (such as Trp in case of BR) introduced specifically in an interactive pocket for carotenoid to protein and retinal. Gly145 of ActR-13023 aligned perfectly with G156 of XR and created space for docking the keto-ring of SAL into the protein (Figure 1C). Phylogenetic analysis suggested that ActR-13023 shared a close evolutionary relationship with XR, which functions as proton-pumping rhodopsin (Figure 1D).

### Functional Expression of ActR-13023

**Figure 2:**
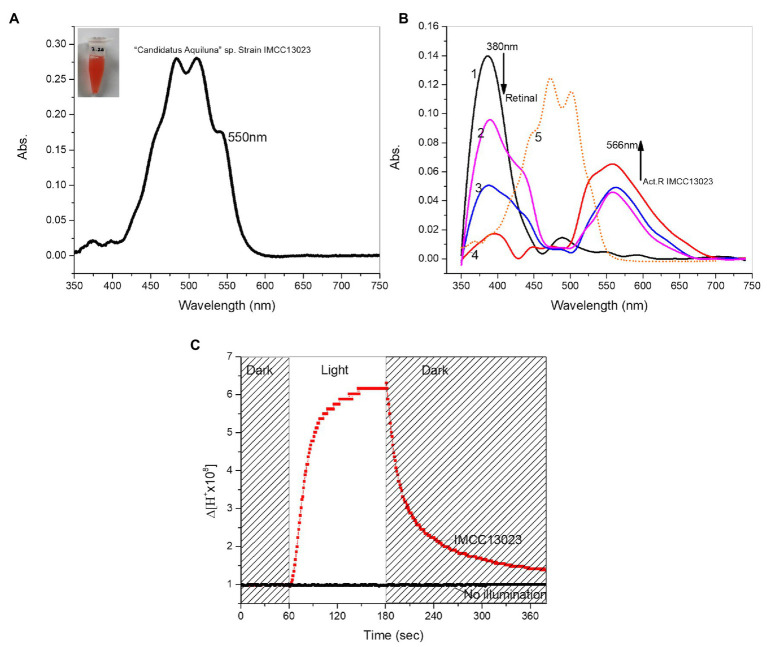
Characterization of ActR-13023 in *Candidatus aquiluna*. **(A)** *C. aquiluna* whole-cell spectrum (black) and expected ActR-13023 peak in the native cell (red). **(B)** Chromophore removal from *C. aquiluna* membrane and reconstitution with all-*trans*-retinal (1) membrane fraction with chromophore removed, (2) 1 h, (3) 24 h, and (4) 48 h after reconstitution, (5) carotenoid isolated from *C. aquiluna*. **(C)** Measurement of proton pumping potential of *C. aquiluna*.

Based on primary and 3D structural modeling, ActR-13023 was predicted to be a light-driven proton pump functioning as a single molecule and assembling with retinal and carotenoids. The function of this protein was studied in native cells. The UV-visible spectrum of native cells showed maximum absorption (λmax) in the blue-green region; λmax was observed at 512 nm and the formation of a blue shoulder peak at 482 nm, a green shoulder peak at 550 nm (Figure 2). To confirm the expression of ActR-13023 in strain IMCC13023, the chromophores were removed from the native membrane using hydroxylamine and reconstituted with all-*trans*-retinal. As anticipated, the absorbance at 566 nm (green region) with a minor shoulder absorption peak formed at 526 nm (blue region) increased upon incubation with all-*trans*-retinal. Chromophore reconstitution confirmed the natural expression of ActR-13023 in native cells. Opsin-bound retinal usually produces a single peak, yet multiple absorbance readings were observed here. After 1 h of reconstitution, the absorbance at 566 nm was increased, then after 2 days of reconstitution, the height of the shoulder peak in the blue region was also increased, suggesting the interaction of carotenoid. As shown in Figure 2B, the membrane of native cells showed minor absorption peaks in the blue region, suggesting that the endogenously synthesized carotenoid was retained in the membrane after retinal removal (Figure 2B graph 1). After 1 h of reconstitution (graph 2), a sharp peak was formed at 566 nm, and after 24 and 48 h (graphs 3 and 4, respectively), the absorbances in the blue region were increased. The carotenoid isolated from the native membrane formed peaks at 472 and 501 nm (Figure 2 graph 5). In this study, the molecule was named “actinobacterial carotenoid” (Figure 2B). The natural expression of ActR-13023 revealed the importance of this protein since the corresponding gene was encoded in the genome even this strain genome is considerably compact (the summed length of the contigs is 1,359,862 bp, the smallest genome size ever reported for free-living actinobacteria, as of April 2012; Kang et al., 2012). The native cells were suspended in unbuffered solution (10 mM NaCl, 10 mM MgSO_4_, and 10 μM CaCl_2_), and light illumination to the sample induced acidification suggesting a light-driven outward proton-pumping activity (Figure 2C). Collectively, the findings indicated that ActR-13023 inherently functions as an outward proton pump for phototrophy.

### Heterologous Expression and Biochemical Characterization of ActR-13023

**Figure 3:**
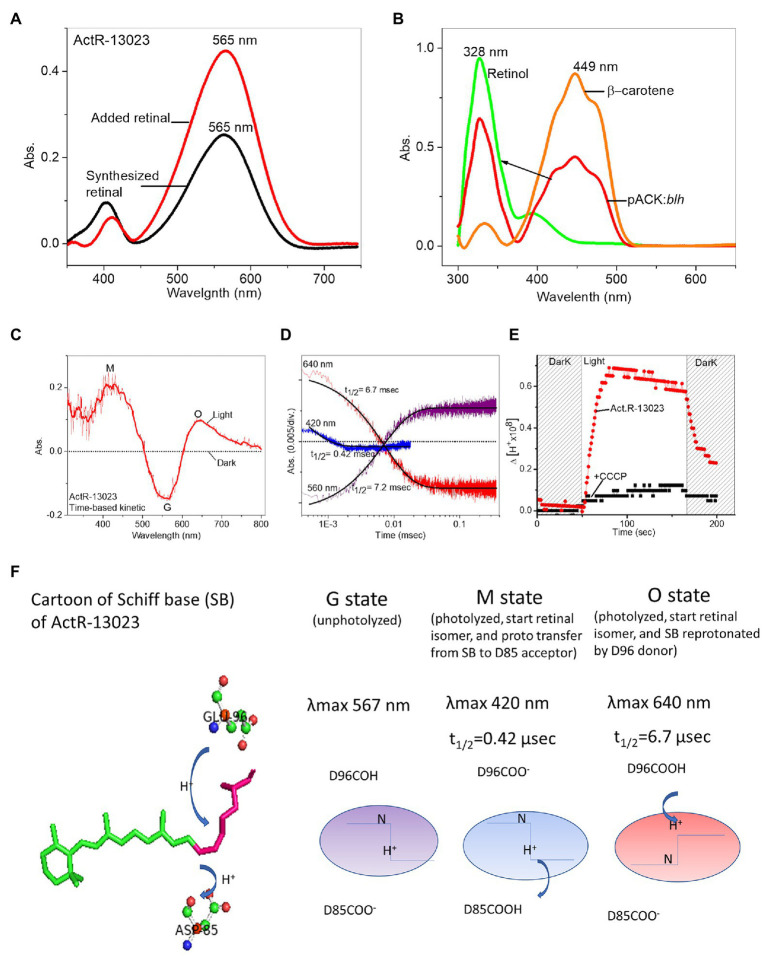
Characterization of heterologously expressed ActR-13023. **(A)** Expression with additional all-*trans*-retinal (red) and bio-synthesized retinal (black). **(B)** Retinol conversion from β-carotene using the enzyme encoded by the *C. aquiluna blh* gene. **(C)** A light-induced differential spectrum of purified ActR-13023. **(D)** Flash-induced transient absorbance changes during the photocycle. **(E)** Proton pumping activity of heterologously expressed ActR-13023. **(F)** Illustration of naturally functional ActR-13023 as a light-driven outward proton pump.

The opsin apoprotein cannot absorb light independently in the absence of a chromophore. Therefore, we hypothesized that the strain IMCC13023 could naturally synthesize retinal as a chromophore for apo-actinorhodopsin (holo-ActR). The *Actinobacteria* genome harbors a bacterio-opsin related protein homolog (*blh*) gene that encodes a β-carotene 15,15′ dioxygenase along with carotenoid synthesis genes and the *actR* gene. Transcription analysis of acI actinobacteria (acI lineage of the phylum Actinobacteria, the most abundant bacterial group in most freshwater lakes; Kang et al., 2017) revealed that all retinal and carotenoid operon genes were transcribed and *actR* was among the most highly transcribed acI genes (Dwulit-Smith et al., 2018). Genome comparison of *Candidatus Aquiluna* sp. IMCC13023 to other acI actinobacteria facilitated identifying the chromophore’s synthesis pathway and retinal-producing β-carotene dioxygenase (accession no. WP_007542589.1), suggesting the presence of the natively synthesized dual chromophores retinal/carotenoid (Supplementary Figures S1A, S2). Heterologously expressed ActR-13023 in *E. coli* cultured in the presence of an exogenously supplemented or endogenously synthesized retinal showed the identical optical spectra with λmax at 565 nm pH = 7.0 when the protein was solubilized in detergent solution (Figure 3A). The *blh* gene from strain IMCC13023 was cloned in a pACK*-blh* plasmid together with other genes encoding proteins participating in the β-carotene-producing pathway. The expression of pAC-BETA (Cunningham et al., 1996) produced β-carotene with maximum absorption at 449 nm (orange). The pigment was treated with β-carotene 15,15′ dioxygenase, which led to the conversion of β-carotene to retinol (red) with λmax at 328 nm; the absorbance of reference retinol was measured for comparison (green; Figure 3B). The co-expression of *actR* and *blh* genes generated functional actinorhodopsin, and light-induced differential spectra of ActR-13023 revealed the light-dependent molecular photochemical changes. Upon light absorption, ActR-13023 entered a photocycle with the formation of several distinct photo intermediates (typical intermediates are M and O) initiated by the isomerization of all-*trans*-retinal to 13-*cis*-retinal. This cycle has been associated with the proton pumping mechanism (Lanyi, 1993). The ActR-13023 light-induced differential spectra showed a clear accumulation of O intermediate with absorption at 645 nm, along with depletion of the ground-state absorption at 560 nm and increase in the M intermediate components with absorption at 420 nm (Figure 3C). Photocycle measurement revealed the rapid formation of kinetic intermediates [M intermediate (t_1/2_ ≈ 0.42 ms), O intermediate (t_1/2_ ≈ 6.7 ms), and return to ground state (t_1/2_ ≈ 7.2 ms)], which is indicative of the highly efficient proton-pumping activity (Figure 3D). ActR-13023-expressed *E. coli* cell showed acidification upon light illumination followed by a dark-light cycle, and the proton pumping activity was abolished upon treatment with carbonyl cyanide m-chlorophenyl hydrazine (Sapra et al., 2003; Figure 3E). Therefore, based on the changes in the differential spectrum and acidification of both native cell and ActR-13023 containing *E. coli*, we concluded that ActR-13023 is a natural light-driven outward proton pump functioning with natively synthesized retinal (Figure 3F).

**Figure 4:**
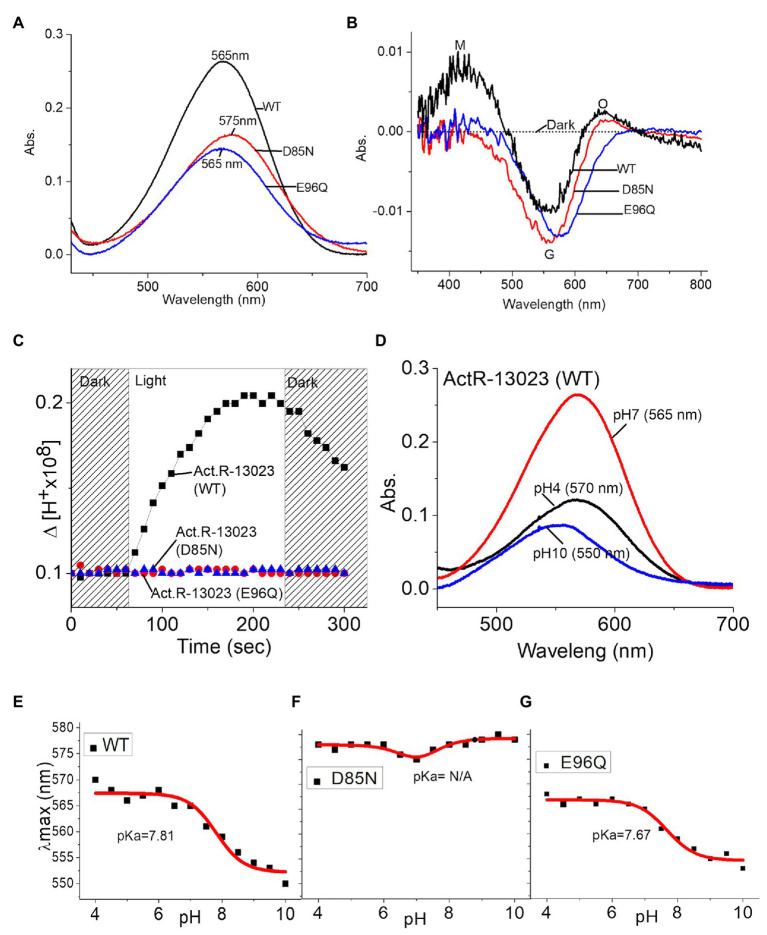
Mutation studies of ActR-13023, wild-type ActR-13023, D85N, and E96Q mutants. **(A)** UV-visible spectra. **(B)** Light-induced differential spectra. **(C)** Proton pumping activities. pH titration and pKa calculation: **(D)** ActR-13023 spectra at pH 4, 7, and 10, **(E)** pKa value of ActR-13023, **(F)** pKa value of the D85N mutant, and **(G)** pKa value of the E96Q mutant.

The high-resolution crystal structure of bacteriorhodopsin revealed the hydrogen bond network’s characteristics and the critical mechanism underlying proton pumping associated with the retinal SB, a proton acceptor (D85), and a proton donor (D96). A mutation in ActR-13023 at the proton acceptor and donor positions inhibited the light-driven proton-pumping activity and significantly altered the protein kinetics. D85N mutant led to the loss of protonation ability from SB. The first essential process for proton transfer from SB to D85 led to the M intermediate. In the case of the D85N mutant, no proton acceptor can be protonated by SB, so no M intermediate was detected in the light-induced spectra. Proton uptake from the proton donor D96 occurred during the transition from the M to O intermediate. When wild-type ActR-13023 was used, the O intermediate absorbed light at 640 nm. However, this absorbance was not observed for the E96Q mutant. Functional mutation studies on ActR-13023 revealed a typical primary proton donor and proton acceptor in the pumping mechanism. The mutants expressed no acidification due to the absence of proton translocation across the membrane, which indicated the central roles of these residues in the hydrogen network and the pumping mechanism. The maximum absorption of ActR-13023 depends on the environmental pH, and pKa of the primary proton acceptor can be calculated in pH titration experiments. The pH-dependent shifting of ActR-13023 absorbance allowed us to calculate the pKa of SB counter-ion residues (D85). ActR-13023 showed maximum absorption at 570 nm at pH 4.0 and 550 nm at pH 10.0. The pKa ≈ 7.81 of SB counter-ion D85 as primary proton acceptor was shown in Figure 4E. In contrast, the pKa of the D85N mutant could not be calculated (notably, the small maximum shifted were observed due to the absence of protonatable acceptor residue), and the pKa for the E96Q mutant was 7.67 (Figure 4).

### ActR-13023 Is a Dual Chromophore Rhodopsin

**Figure 5:**
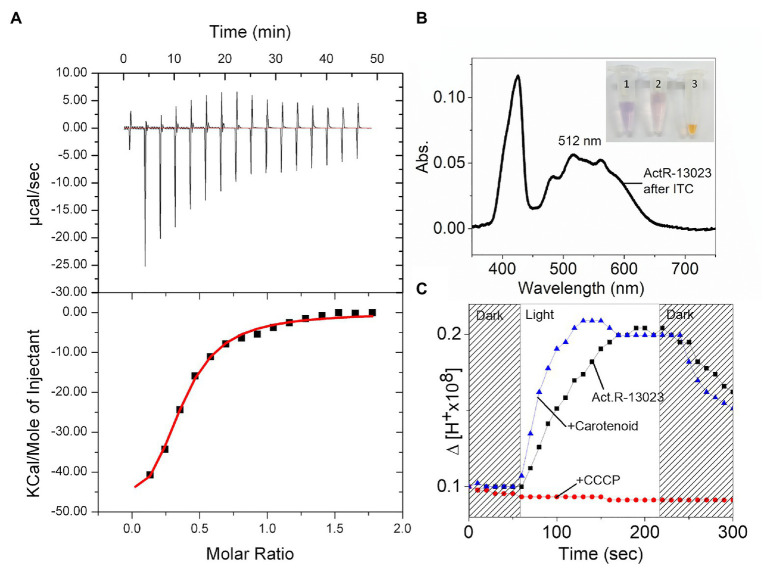
Interaction between ActR-13023 and actinobacterial carotenoid. **(A)** Isothermal titration calorimetry (ITC) of ActR-13023 and the carotenoid. **(B)** UV-Vis spectrum of ActR-13023 after ITC experiment; the samples were shown in e-tube (1) purifiedActR-13023, (2) ActR-13023 after ITC experiment with carotenoid, and (3) the carotenoid as the ligand. **(C)** Comparison of proton pumping in the presence, absence of the carotenoid, and ActR-13023 treated with carbonyl cyanide m-chlorophenyl hydrazine (+CCCP).

The proton pumping activity of heterologously expressed ActR-13023 is comparable to that of other proton-pumping rhodopsins. Proteorhodopsin (PR) was expressed, and the protein expression level and proton pumping of the two rhodopsins were shown in Supplementary Figure S5. Interestingly, the native cells exhibited more buffer acidification in similar light sources and optical cell density at 600 nm. The orange color of the native cell and carotenoid-related genes in the genome suggested carotenoids’ expression in this strain. Like, XR from *S. ruber* that assembles with salinixanthin as a secondary light-harvesting antenna, ActR-13023 was expected for the possibility of carotenoids binding, which may functionally maximize its potential. A carotenoid molecule isolated from the strain showed a molecular weight of 591.4946 mz, and the absorption peaks were formed at 472 and 501 nm in the UV-visible spectrum (Figure 2B, graph 5). Thermodynamic insights from isothermal titration calorimetry experiments indicated a significant interaction between ActR-13023 and the carotenoid. The isolated carotenoid was titrated against ActR-13023 (as a ligand and receptor), and the essential enthalpy changes were measured after every injection. The titration profile could be best fitted using a nonlinear least-squares approach to the “one set of sites” binding model, which yielded the association constant (K_a_), the stoichiometry of binding (n), thermodynamic parameters, enthalpy of binding (ΔH), and entropy of binding (ΔS) values. Measurements at 30°C yielded the following values for K_a_, n, ΔH, and ΔS: 2.72 (±0.8) × 10^4^ M^−1^, 0.751 (±0.03), −6.72 × 10^4^ (±0.83) cal.mol^−1^, and −201 cal.mol^−1^.deg.^−1^, respectively, and the calculated changes in Gibb’s free energy (ΔG) was −6.26 kcal/mol^−1^ (Figure 5A). After the isothermal titration calorimetry experiment, the samples were speculated by UV-VIS spectrometer, and the absorption spectrum showed carotenoid binding characteristic where maximum absorption was observed at 512 nm accompany the shoulder shape (Figure 5B). These results indicated that purified ActR-13023 could be reconstituted with isolated carotenoids. We tried to express ActR-13023 by exogenously added the carotenoid. However, after purification, no binding complex could be isolated. This indicated that the carotenoid barely penetrated the cellular membrane, so the carotenoid was added during spheroplast preparation (see Materials and Methods section) to maximize the possibility of penetration, and samples were incubated overnight for the reaction. The absorption spectra of spheroplast showed optical shifts owing to carotenoid engagement. The ActR-13023 spheroplast showed maximum absorption at 557 nm, which shifted to 516 nm upon carotenoid binding. The addition of carotenoids gave no significant enhancement to total pH change in buffer acidification but notably improved the initial pumping rate of ActR-13023 (Figure 5C). In contrast, when salinixanthin was added to the samples, we did not see the enhancement, but a lower proton pumping rate was observed (Supplementary Figure S5). This gave an interesting question about carotenoid interaction with rhodopsin and its impacts on protein function. However, further study is required to answer this phenomenon.

## Discussion

The high abundance of actinobacteria in aquatic habitats, seawater, sea ice, and freshwater has led to establishing a cellular adaptation model for energy scavenging fitness. The actinorhodopsin-encoding gene has been detected in the compact genome of *Candidatus Aquiluna* sp. IMCC13023, along with the retinal and carotenoid biosynthesis genes. Our results validated the importance of ActR in actinobacteria for solar energy capture and indicated its utility under nutrient stress. The biosynthetic pathways of retinal and its carotenoid precursors have been reported based on findings from the assessment of single-cell genome and metagenome sequences of freshwater-isolated actinobacteria categorized in the acI lineage clades A and B (Dwulit-Smith et al., 2018). Unlike other bacteria that encode opsins but may acquire retinal exogenously, actinorhodopsins had their chromophore synthesis machinery.

Interestingly, ActR-13023 is natively expressed with retinal and interacts with natively synthesized carotenoids as secondary chromophores. Seven genes in the actinobacteria genome are considered to encode proteins associated with retinal synthesis. Other genes that encode proteins for the synthesis of complex carotenoids, such as the β-carotene ketolase gene, have also been detected (Klassen, 2010). Consequently, ActR-13023 might be bound to retinal and complex carotenoids. The 4 keto-ring of complex carotenoids salinixanthin, canthaxanthin, and echinenone have been reported to be critical components for rhodopsin binding (Balashov et al., 2010). However, the co-expression of ActR-13023 and canthaxanthin in *E. coli* did not lead to the production of a binding complex (data not shown). We also stimulated ActR-13023 with canthaxanthin for evaluating its binding properties. Molecular docking studies suggested that the docking surface was in a position like that in XR; binding with salinixanthin was promoted; however, the binding constant could not be estimated (Supplementary Figure S1C). The carotenoid’s molecular weight isolated from the strain showed 591.4946 in molecular mass, which was greater than those of lycopene (536.8) and canthaxanthin (564.8), suggesting a distinct structure of this carotenoid. AcI actinobacterial gene transcription has been challenging to study. While the isolation of several carotenoid-related genes from the strain had been attempted, but the carotenoid had been unsuccessful. Light-driven outward proton pumping by native cell was significantly stronger than that in ActR-13023-overexpressed *E. coli*, which suggested the carotenoid’s critical role for light-driven proton pumping by ActR-13023. Molecular docking results suggested the interaction of canthaxanthin containing helices E, F, and G. However, the titration of canthaxanthin with purified ActR-13023 and co-expression did not show the binding property. This suggested that despite a similar binding pocket on helices E and F, microbial rhodopsin might exhibit selectivity toward secondary chromophores. The carotenoid from the strain may contain a unique structure compatible with ActR-13023. It will be interesting to elucidate the mechanism of dual chromophores (retinal/carotenoid) in actinorhodopsin. Our results showed that the actinobacterial carotenoid barely penetrated the cell membrane; therefore, the secondary antenna might be synthesized within the cell, or a specific carotenoid uptake mechanism may be acquired to obtain it *via* exogenous sources. Notably, ActR-13023 followed a rapid photocycle with the return to the ground state with a half-time of 7.2 msec. It exhibited proton pumping activity comparable to that of other well-known proton pumps such as proteorhodopsin. The potential of actinorhodopsin with dual chromophores in the aquatic environment on the earth’s surface may play a critical role in solar energy capture. Effective solar bio-energy conversion may serve as a helpful strategy against climate change issues, besides having other applications, and the role of ActR for solar energy capture in microbial communities could be the contribution to the ecosystem.

## Data Availability Statement

The original contributions presented in the study are included in the article/Supplementary Material, further inquiries can be directed to the corresponding author.

## Author Contributions

KC and K-HJ developed the concept and supervised the experiments. SK provided support in the cloning and plasmid preparation experiments. J-gS, S-GC, K-WK, J-HK, SM, and H-SC provided support in the protein expression and purification experiments. All authors contributed to the article and approved the submitted version.

### Conflict of Interest

The authors declare that the research was conducted in the absence of any commercial or financial relationships that could be construed as a potential conflict of interest.

## Footnotes

^1^www.dockingserver.com
