# Supplementary material for: Assembly of Natively Synthesized Dual Chromophores Into Functional Actinorhodopsin
Source: Front Microbiol. 2021 Apr 28;12:652328. doi: 10.3389/fmicb.2021.652328 (PMC8113403; doi:10.3389/fmicb.2021.652328)
Supplement: Supplementary file 1 [file Data_Sheet_1.PDF]

frontiers

Supplementary Material

A

Primer names	Sequences (5' to 3')	Target genes	Accession
ActR-13023 forward	GCACATATGAGCTATGAAGG	Actinorhodopsin (ActR-13023)	WP_040324815.1
ActR-13023 reverse	GCAGCGGCCGCCTACTTTGAAACTTCTTC		
IMCC13023-blh forward	GCACATATGGTGACTTCCGAGATAAG	beta-carotene 15,15'-dioxygenase, Brp/Blh family	WP_007542589.1
IMCC13023-blh reverse	GCAGCGGCCGCCTAGGGCAACACCGAATTTTTTG		
PCC7120-ctrW forward	GCACATATGGTTCAGTGTCAACCATCATC	CrtW beta-carotene-ketolase	WP_010997340
PCC7120-ctrW reverse	GCAGCGGCCGCTTATAAAGATATTTTGTGAGC		
IMCC13023-HeR forward	GCACATATGCATCATCACCATCACCACGCAAAAACA ATAAAGCCGGC	Heliorhodopsin HeR	WP_007540014
IMCC13023-HeR reverse	TGCGCGGCCGCTTAGCTGACCAAAGCCGG		
IMCC13023-ctrY123 forward	GCACATATGCTTTTATTCGTG	lycopene cyclase domain-containing protein	WP_007542581.1 WP_007542583.1 WP_007542586.1
IMCC13023-ctrY123 Reverse	GCAGCGGCCGCCTATTTTTTCCGTTTGGC		
IMCC13023-ctrIEY Forward	GCACATATGCAGCTCGGCTAC	Putative carotenoid synthesis related genes	EIC91895.1 EIC91896.1 EIC91897.1
IMCC13023-ctrIEY Reverse	GCAGCGGCCGCTTAGCTTGGACCTATAAG		

B

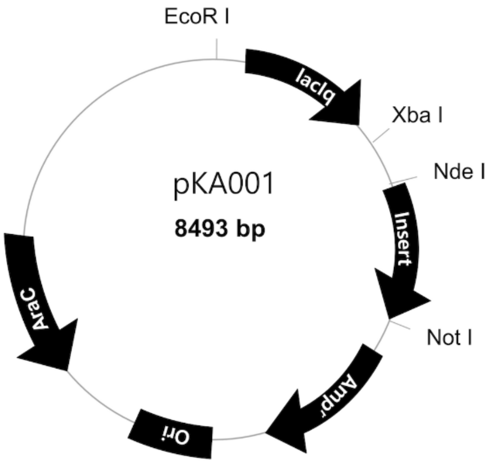

C

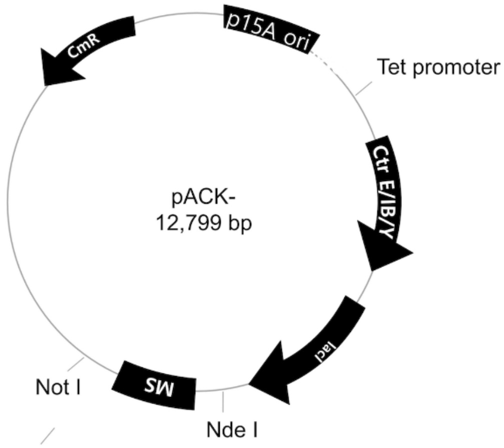

Supplementary figure 1. Primers sequences and global map of the expression vector. (A) The primer's name, oligomer sequence from 5' to 3', its target genes, and the accession number. (B) PKA001 vector was used for opsin expression. (C) pACK- vector map is a modified pAC-BETA plasmid with an additional multiple cloning site with an inducible promoter for retinal and carotenoid-related genes study.

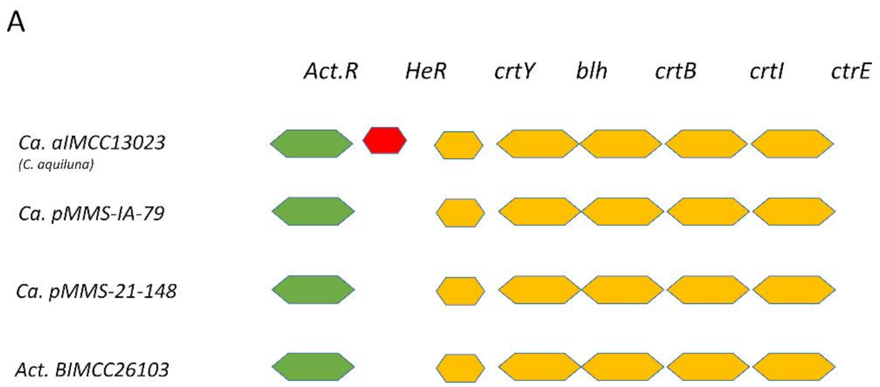

B

```
Ca.AIMCC13023      MLESQLRLLVQLRTVSRYSIFAGIIISIPLN-ILLPHSISWQVVLAITALAIGIPHGAVD      59
Ca.pMMS-IA-79      MSETKLRLFSRVRTLSSAVIAIATVLSLLFSQWLGTNSLGWQLVLATIALAIGIPHGALD      60
Ca.pMMS-21-148     MREIELDLLLKVRKYSRYAVGVAIVLSFAFARIIEESEITWQVVIALIALAIGIPHGALD      60
Act.BIMCC26103     MAEIELDLFLKVRKFSRIAVAVTIVATFIFAQVIASSDTTWQVVIAVIALAAGIPHGALD      60
                   * * :* *: ::*. *    :     : :: :    :    .  **:*:*  *** ******:*

Ca.AIMCC13023      HLITVPKFKFFKMALFLIGYLTVTGLCIWFILSNNLIGFQLIVLISALHFGIGDASFISE      119
Ca.pMMS-IA-79      HLVTLPKAAPIRMALFIMLYVAIALLAIYAILRWNVWGFIFVVIMSATHFGIGDAAFLSE      120
Ca.pMMS-21-148     HLVTLPRSNFKKMALFITLYVAVAVLAVIALLTWNVVGFILVVIMSAVHFGIGDAAFISE      120
Act.BIMCC26103     HLVTLPKSSSLKMAGFIAIYVAVAVIAVIALLTWNVAGFIAVVAMSAVHFGIGDAAFISE      120
                   **:*:*:    :** *:  *:::: :.:  :*  *: **  :* :** *******:*:**

Ca.AIMCC13023      MDARS-NRTGFPKVLFALAAGFTPVFIPLLNSRSTEALETVNPILSGWANPITEQLFWVV      178
Ca.pMMS-IA-79      KDSLD-GSSRIPAWFYAPAAGLLPVAIPLVNSRSTDALEKVNPALINWHSGFTTEILMAV      179
Ca.pMMS-21-148     IDRRSEETKRFQKYLYATAAGTLPVVIPLVSDKSTSALEKVNPALVDWHQGLNNDLMLWV      180
Act.BIMCC26103     IDRRSTDPKPFPRYLYALAAGTLPVVIPLVSDKSAGALERVNPALLNWHHGLNNDLLLWT      180
                    *  .   . :   ::* ***  ** ***:..:*: *** *** * .*   :. :::  .

Ca.AIMCC13023      VTLNLFVTSVMLFKGRRAEAIDLAALLAISLIAPPLVAFAFYFGFWHALRHTGRLTLELP      238
Ca.pMMS-IA-79      AVVTTLCLLALLQRKRYRDALDILLLAALASFAPPLVAFAVYFGCWHAMRHTARLTSLLP      239
Ca.pMMS-21-148     MLITAFALLRLVQRRRDGEAIDLVLLYLLAVTAPPLVAFAVYFGCWHAMRHTARLTLTLP      240
Act.BIMCC26103     MLLTAIALLRLVQKRRDAEAIDLVLLYLLAITAPPLVAFAVYFGCWHAMRHTARLTLVLP      240
                     :. :    :: : *  :*:*:  *  ::  ********.*** ***:***.***  **

Ca.AIMCC13023      SSIKAHERQKPLRAFWLAVAAGLPALAIVIGFTVVLGITGNFDLGADLLWLLLAVVWALT      298
Ca.pMMS-IA-79      NSESAYLRGRPGQAFVAAVIPGLPALVGTLIFVVILAGFSQQDLSDKFLWLTLVTIWALT      299
Ca.pMMS-21-148     SSQEAFTNGSAKRAFIRAVLPGTPALVGTFVIAALIVLLRGDSLDDQFLWVSLVVVWALT      300
Act.BIMCC26103     RSQKAFTEGNSGKAFLGAIWPGLPALVGTFIVACGIIIFRRDSLSDQFLWITLVVVWALT      300
                    * .*. .    :**  *:  * ***. .: ..  :      .*. .:**: *..:****

Ca.AIMCC13023      IPHMALTSRLDAKAMGFSKKKSVVNTN 325
Ca.pMMS-IA-79      VPHMIVTARLDRAALKK---------- 316
Ca.pMMS-21-148     VPHMAVTARLDRKALN----------- 316
Act.BIMCC26103     VPHMAVTARLDRKALS----------- 316
                   :*** :*:***  *:
```

Supplementary figure 2. ActR-13023 and actinobacterial carotenoid. (**A**) Genome region's comparison of *Candidatus aquiluna sp*. Strain IMCC13023's opsin and chromophores synthesis related genes to the transcription study of acl *Actinobacteria* clade A, B, C by Dwulit-Smith et al., 2018. HeR is recently annotated heliorhodopsin gene. The gene was cloned but not expressed in *E. coli*, and the study of this HeR is out of our current study scope, but since the gene was neighborly located, we place it in a red mark for note. The accession number to each gene also documented along with the primers' sequence in this study. (**B**) Beta-carotenoid dioxygenase genes comparison among acI *actinobacteria*.

A

Rank	Est. Free Energy of binding	Est. Inhibition Constant, KI	vdW+ Hbond + desolv	Electrostatic Energy	Total Intermolec. Energy	Frequency	Interact. Surface
1.	131.22 kcal/mole	N/A	+124.69 kcal/mole	+0.09 kcal/mole	+124.78 kcal/mole	50%	1016.49

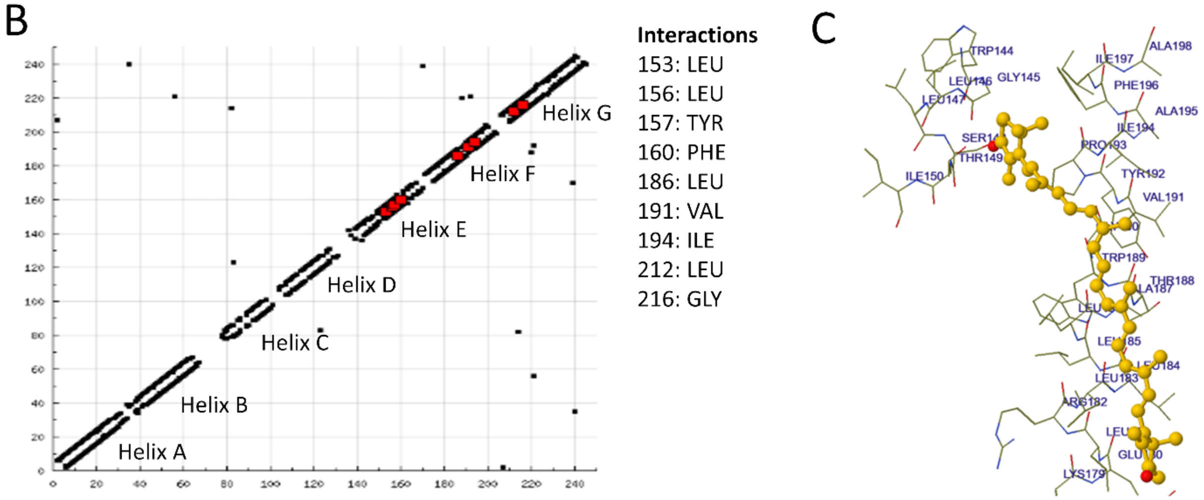

Supplement figure 3. Molecular docking of ActR-13023 to canthaxanthin. (A) Summary result of docking calculation. (B) HB plot of protein and ligand, each black dot represents residues, which aligned to 7 helical structure shown from helix A to G. The main interactions shown in red and position and residues was named on the right. (C) Cartoon structure of ActR-13023 and canthaxanthin interaction residues and their surrounding residues. Docking calculations were carried out using DockingServer (Bikadi and Hazai, 2009). Affinity (grid) maps of 20×20×20 Å grid points and 0.375 Å spacing were generated using the Autogrid program (Morris et al., 1998).

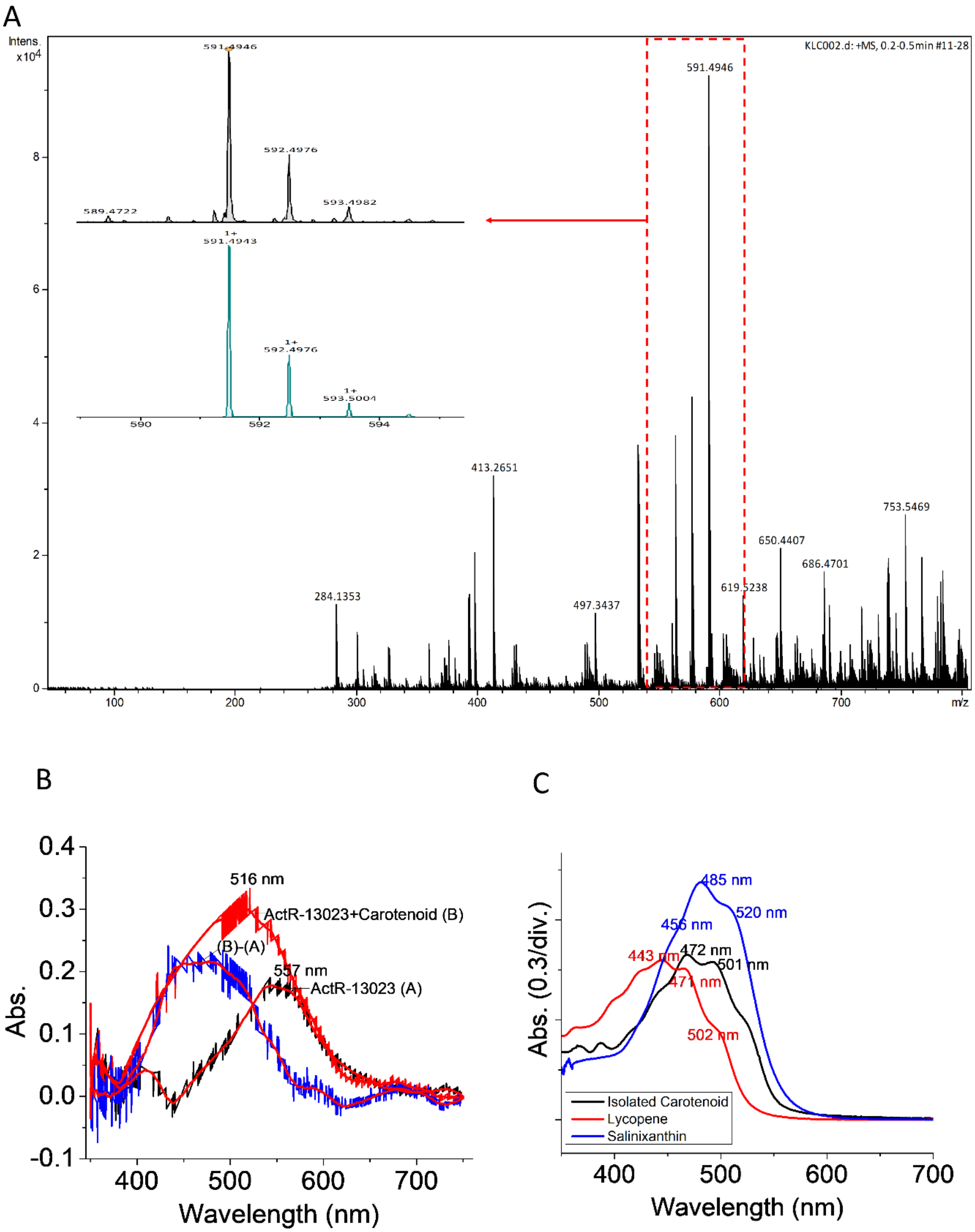

Supplementary figure 4. Actinobacterial carotenoid and Act-13023 spheroplast reconstitution. **(A)** Mass-spectroscopy of isolated carotenoid from *C. aquiluna*. (**B**) UV-visible spectra of Act-13023 spheroplast with and without actino-carotenoid. (**C**) UV-visible spectra of isolated carotenoid (black), lycopene (red), and salinixanthin (blue).

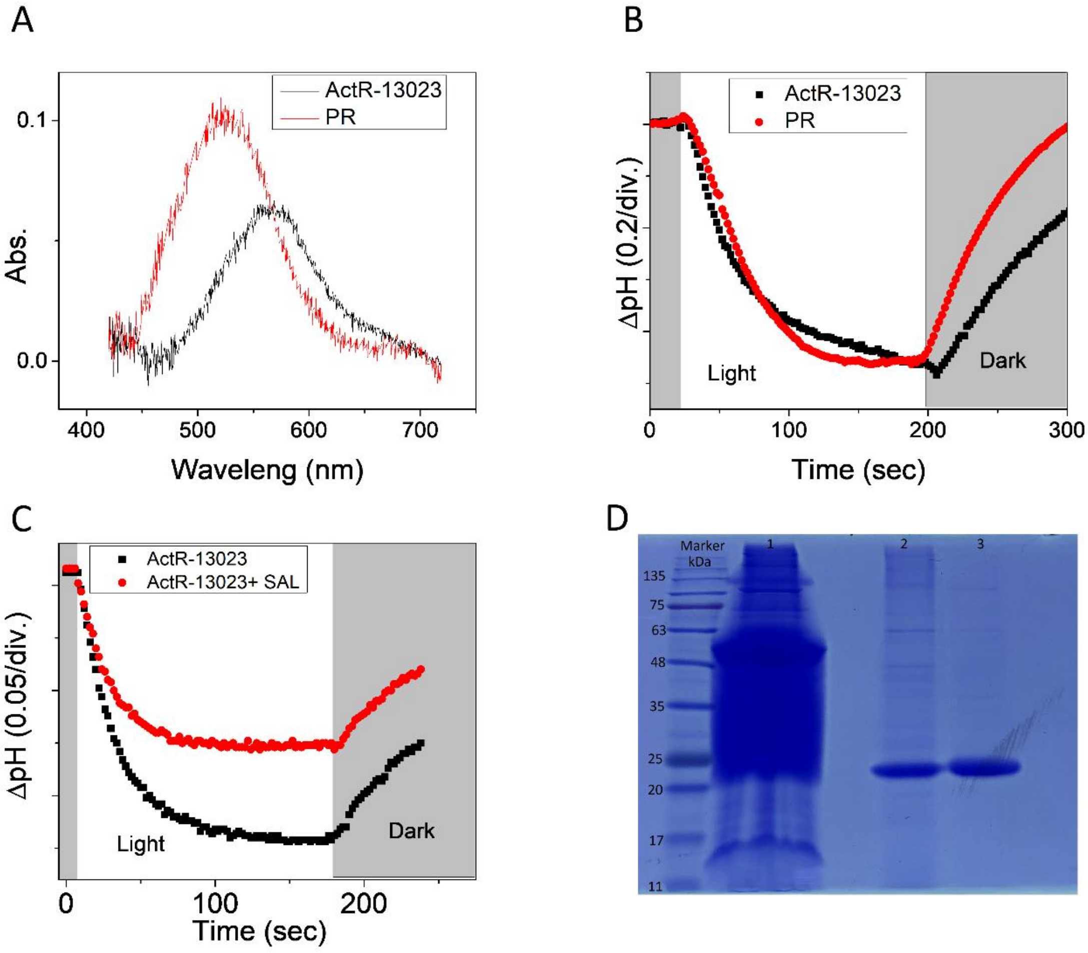

Supplement figure 5. Proton pumping activities of ActR-13023 with Proteorhodopsin (PR), addition of Salinixanthin (SAL), and SDS-PAGE of purified samples. (A) ActR-13023 and PR expressed *E. coli* cells. (B) Proton pumping of the whole cell-expressed ActR-13023 and PR (3 ml of O.D $_{600\ nm}$=1.0 of both samples were used). (C) Proton pumping of ActR-13023 with and without the addition of salinixanthin. In salinixanthin, the proton pumping activity was reduced, while a different effect was observed in *C. aquiluna* extracted carotenoid. (D) SDS-PAGE of ActR-13023: (1) *E. coli* membrane fraction, (2) eluted sample in immobilized metal affinity chromatography (IMAC) process, (3) purified sample after Amicon filter.

References

Bikadi, Z., and Hazai, E. (2009). Application of the PM6 semi-empirical method to modeling proteins enhances docking accuracy of AutoDock. *J Cheminform* 1, 15.

Dwulit-Smith, J. R., Hamilton, J. J., Stevenson, D. M., He, S., Oyserman, B. O., Moya-Flores, F., et al. (2018). acI Actinobacteria Assemble a Functional Actinorhodopsin with Natively Synthesized Retinal. *Appl. Environ. Microbiol.* 84.

Morris, G. M., Goodsell, D. S., Halliday, R. S., Huey, R., Hart, W. E., Belew, R. K., et al. (1998). Automated docking using a Lamarckian genetic algorithm and an empirical binding free energy function. *Journal of Computational Chemistry* 19, 1639–1662.
